# Treatment Resistance in Severe Asthma Patients With a Combination of High Fraction of Exhaled Nitric Oxide and Low Blood Eosinophil Counts

**DOI:** 10.3389/fphar.2022.836635

**Published:** 2022-04-20

**Authors:** Yuki Hoshino, Tomoyuki Soma, Yoshitaka Uchida, Yuki Shiko, Kazuyuki Nakagome, Makoto Nagata

**Affiliations:** ^1^ Department of Respiratory Medicine, Saitama Medical University, Saitama, Japan; ^2^ Allergy Center, Saitama Medical University, Saitama, Japan; ^3^ Research Administration Center, Saitama Medical University, Saitama, Japan

**Keywords:** blood eosinophil count, fraction of exhaled nitric oxide (feno), sputum eosinophilia, sputum neutrophil-predominant, asthma exacerbation (AE), anti-IL-5 biologics, omalizumab (xolair), severe asthma

## Abstract

**Background:** Combining a fraction of exhaled nitric oxide (FeNO) and blood eosinophil count (B-EOS) may be a useful strategy for administration of biologics such as anti-IgE or anti-IL-5 to patients with type 2 inflammatory-predominant severe asthma and is important to be elucidated considering the increasing use of biologics.

**Methods:** This cross-sectional study analyzed the clinical data from 114 adult patients with severe asthma, who were treated at Saitama Medical University Hospital. The eligible patients were stratified into four subgroups defined by thresholds of FeNO and blood eosinophil (B-EOS) counts to detect sputum eosinophilia, using the receiver operating characteristic curve analysis. A total of 75 patients with optimal samples were stratified into four subtypes defined by thresholds of sputum eosinophilia and neutrophilia. Clinical characteristics, pattern of biologics, and distribution of sputum subtypes were analyzed in the stratified subclasses according to the FeNO and B-EOS thresholds. The asthma exacerbation (AE)-free time of the FeNO/B-EOS subgroups and any biologics treatment including anti-IgE or anti-IL-5 use were examined using the Kaplan–Meier method. The hazard ratios (HRs) for AE-free time were examined using the Cox proportional hazard model.

**Results:** The optimal cutoff values for prediction of sputum eosinophilia were defined as ≥2.7% wherein for FeNO as ≥27 ppb and B-EOS as ≥265/µL were considered. The high-FeNO subgroups showed significant high total IgE, compared with the low FeNO. The high-FeNO/high-B-EOS and the high-FeNO/low-B-EOS subgroups showed the largest prevalence of mepolizumab and benralizumab use among the other FeNO/B-EOS, respectively. The high-FeNO/low-B-EOS showed the largest frequency of AEs, high HR, and the shortest AE-free time, among the other FeNO/B-EOS. The sputum eosinophil-predominant subtype was the great majority in the high FeNO/high B-EOS. A diverse distribution of sputum leukocyte-predominant subtype was observed in the other FeNO/B-EOS. The subsequent AE-free time and its HR were comparable among the biologics use groups.

**Conclusion:** The strategy of classifying severe asthma based on the combination of FeNO and B-EOS proposes particular refractory type 2 severe asthma and underlying airway inflammation as a feasible trait for optimal biologics use.

## Introduction

Several recent reports have demonstrated that patients with severe asthma characterized by a type 2 inflammatory-predominant endotype are a treatable population (Agache et al., 2020; Holguin, et al., 2020; Hammad and Lambrecht, 2021; Buhl, et al., 2021). Randomized clinical trials and further meta-analyses of several approved biologics, including anti-IgE, anti-IL-5, and anti-IL-5α receptor, and more recently anti-IL-4α receptor antibodies have demonstrated that they are clinically beneficial to patients with the type 2 inflammatory-predominant endotype (Farne, et al., 2017; Agache et al., 2020; Ramonell and Iftikhar, 2020; Bousquet, et al., 2021). The use of anti-type-2-inflammation biologics resulted in reduced rates of asthma exacerbation (AE) and improved pulmonary function and disease control. Several studies have surveyed predictors of responders or super responders to biologics, including blood eosinophil counts (B-EOS), fractional exhaled nitric oxide (FeNO), and history of previous asthma exacerbation (Hanania, et al., 2013; Ortega, et al., 2016; Casale et al., 2019; Harvey, et al., 2020; Kavanagh, et al., 2020; Bleecker, et al., 2020; Kroes et al., 2020; Jackson et al., 2021; Kavanagh, et al., 2021; Eger, et al., 2021). Although the use of biologics to treat patients with type 2 inflammatory-predominant severe asthma is becoming more popular, it does not necessarily help to attain a well-controlled condition (Nagase et al., 2020; Jackson et al., 2021). The candidate biomarkers that would facilitate the prediction of a satisfactory response have been explored, but they are generally varied and are to be fully elucidated yet.

B-EOS and FeNO are recognized as representative biomarkers of type 2 inflammation in asthma. Notably, the Global Initiative for Asthma (GINA) report of 2019 recommends both as biomarkers that are indicative of type 2 inflammatory-predominant severe asthma (Global Strategy for Asthma Management and Prevention, 2021). B-EOS can potentially cause eosinophilic airway inflammation (Salter and Sehmi, 2017), while FeNO is an inflammatory product delivered from the bronchial epithelium and is mediated mainly by IL-13 (Chibana, et al., 2008; Matsunaga et al., 2021). Both type 2 inflammatory biomarkers can predict eosinophilia in the airway and are recognized as risk factors for AEs (Dweik, et al., 2010; Malinovschi, et al., 2013; Schleich et al., 2014; Korevaar, et al., 2015; Price, et al., 2015; Denlinger, et al., 2017; McDowell, et al., 2021; Busse, et al., 2021; Matsunaga et al., 2021; Soma, et al., 2018). Furthermore, using them in combination has been shown to enhance the risk ratio of AEs (Malinovschi, et al., 2013; Soma, et al., 2018; Kraft, et al., 2021; Busse, et al., 2021). The two-dimensional classifications defined by these biomarkers demonstrated clinically distinctive features in four subgroups and exhibited differences in sensitized allergen numbers and prevalence of comorbid sinusitis, magnitude of response to oral corticosteroid (OCS), trends of incremental odds ratios of AEs, and progress of severe airflow limitation and airway hyperresponsiveness (Malinovschi, et al., 2016; Oishi et al., 2017; Soma, et al., 2018). Given these reports, the FeNO- and B-EOS-based classification is expected to stratify the selection of use of biologics. However, there is no evidence yet correlating the use of biologics to the B-EOS/FeNO classification of patients with severe asthma.

Comprehensive analysis, including genomics of bronchial epithelial cells and inflammatory cells in sputum and leukocytes, cytokines, and chemokines in sputum have advanced our understanding of the endotypes of severe asthma (Moore, et al., 2014; Hinks et al., 2016; Takaku et al., 2016; Fricker et al., 2019; Peters, et al., 2019). Clusters of the predominant-granulocytic type in sputum identified particular appearances, showing tolerance against OCS, upper airway comorbidities, fixed airflow restriction, poor symptoms, and an increase of FeNO (Hinks et al., 2016). However, more information is required to help this distribution profile leverage of the B-EOS- and FeNO-based four subgroup classifications for the maximal therapeutic advantage.

This study aimed to determine whether the stratification of patients with severe asthma according to type 2 biomarkers (B-EOS and FeNO) profiled trends of biologics use, and whether these trends for patients with type 2 inflammatory-predominant severe asthma and their clinical efficiency differed from real-world practice. Distribution of subclasses of airway inflammation based on eosinophil and neutrophil counts in sputum was also determined under those type 2 biomarker subclasses. We finally determined whether any particular aspect was associated with the efficiency of biologics.

## Materials and Methods

### Study Design and Participant Selection

This retrospective cohort study was conducted to determine the characteristics and specific use of biologics in patients with severe asthma who were classified based on a combination of FeNO and B-EOS counts and their efficiency of responses to biologics. The enrolled patients and their clinical data from the previous or ongoing registries of observational studies at our institution (approval numbers: 13-083, 14-009, 19-132, 788, and 789) were included in this study. The Institutional Review Board of Saitama Medical University Hospital approved this study (approval number: 2021-096). Written informed consent was obtained from all participants.

The participants were more than 20 years old and attended the Allergy Center and the Department of Respiratory Medicine of Saitama Medical University Hospital from 2012 to 2021. The participants were enrolled from 1 December 2013 to 30 November 2020. Asthma diagnosis was performed according to the Japanese Society of Allergology guidelines (Nakamura et al., 2020). The positively identified patients had typical symptoms of asthma or episodes of exacerbations and ≥12% reversibility of the predicted value of FEV1 and/or airway hyperresponsiveness (PC20 methacholine <8 mg/ml). Severe asthma was diagnosed according to the American Thoracic Society/European Respiratory Society (ATS/ERS) guidelines (Chung, et al. 2014). High-dose inhaled corticosteroids and a long-acting β-agonist (LABA), leukotriene modifier (LTRA) or slow-sustained theophylline of the previous year, or oral corticosteroids for more than 50% of the past year were required to control asthma. Diagnoses of upper airway diseases and allergic dermatitis were basically carried out by an otolaryngologist or a dermatologist or based on their clinical history. The patients with the following indices were excluded: the presence of comorbidities, which might modify the results in this study, including allergic bronchopulmonary aspergillosis, eosinophilic pneumonia, eosinophilic granulomatosis with polyangiitis, and hypereosinophilic syndrome; respiratory infection for 4 weeks prior to the day of examinations; pregnancy; and the presence or history of malignant tumors, severe renal failure, or severe heart failure.

All the participants had undergone a blood examination, pulmonary function, and FeNO assessments, and some of them had induced sputum in some of our other studies. Medical information was also obtained from those studies. Spirometry was performed using an AS307 spirometer (Minato Medical Science, Osaka, Japan) (Miller et al., 2005). FeNO was measured using a compact device (NIOX VERO, Circassia AB, Oxford, United Kingdom) prior to spirometry, according to the recommendations of the ATS/ERS (American Thoracic Society and European Respiratory Society, 2005).

The incidents of AE over 12 months since the first date of entry were summed up. The first day for the patients treated with biologics was defined as the day of biologics initiation. According to the ATS/ERS guidelines, the need for hospitalization or two or more courses of OCS is referred to as a severe asthma exacerbation (SAE) (Reddel, et al., 2009). Recurrent bronchodilator use for rescue for two or more days because of worsening of symptoms and/or lung function or any SAE appearance is defined as AE.

We determined the optimal values of eosinophil and neutrophil ratios in the sputum of patients with asthma for the classification of airway eosinophilia and neutrophilic dominance. Subsequently, we determined the optimal predictive values of FeNO and B-EOS counts for sputum eosinophilia defined as the derived value of the sputum eosinophil (Sp-EOS) ratio.

### Induction of Sputum and Processing

The induction of sputum was performed, as described previously (Fahy, et al., 2001; Paggiaro, et al., 2002). Briefly, the participants inhaled sterile hypertonic saline (4.5%) using an ultrasonic nebulizer (MU-32, Azwell, Osaka, Japan) after inhalation of salbutamol using a metered-dose inhaler. After they rinsed their mouths, they unforcedly expected sputum to some extent at 5-min intervals for up to 20 min.

The sputum samples collected in 50 ml polypropylene tubes were kept at 4°C and processed within 2 h as follows: first, 1 ml of Hank’s balanced salt solution containing 1% dithiothreitol (Sigma, St. Louis, MO., United States) was added to the sputum samples. Next, the admixture was vortexed and gently aspirated at an ambient temperature to homogenize the sputum samples. Finally, HBSS was added up to 5 ml and the samples were then centrifuged at 400 × g for 10 min. The supernatants were stored at −80°C until further examination. Cell pellets were resuspended in phosphate-buffered saline. Cytospin slides (Cytospin 3: Shandon, Pittsburgh, Pennsylvania) of the resuspended cells were prepared and stained with May–Grunwald–Giemsa stain. An independent investigator counted more than 500 cells per slide. Squamous cells <61% in the samples was considered appropriate for the analysis (Uchida et al., 2019).

### Determination of Clinical Characteristics and Particular Uses of Biologics in Type 2 Inflammatory Subclasses Classified Based on FeNO and B-EOS Counts

We determined the optimal predictive values of FeNO and B-EOS counts for sputum eosinophilia defined as the derived value of Sp-EOS ratio. We evaluated the sensitivity and specificity of FeNO and B-EOS counts for their diagnostic accuracy. Based on the optimal cutoff values of FeNO and B-EOS counts patients with asthma were classified into four type 2 inflammatory subclasses herein referred to as low FeNO (FeNO^lo^)/low B-EOS (B-EOS^lo^) subclass, high FeNO (FeNO^hi^)/B-EOS^lo^ subclass, FeNO^lo^/high B-EOS (B-EOS^hi^) subclass, and FeNO^hi^/B-EOS^hi^ subclass.

We evaluated the characteristics of these patients in terms of demographics, AEs, pulmonary function, B-EOS counts, blood neutrophil counts, serum total IgE, FeNO, Sp-EOS and neutrophil (Sp-NEU) ratios, and distribution of the four airway-inflammatory subtypes. We also determined specificity of biologics use according to the subclass. We determined if there were differences in number of annual occurrences and time to subsequent AE and SAE prior to and post-use of biologics.

### Deviation of Airway-Inflammatory Subtypes Based on Deviation of Sputum Granulocytes

Thresholds of sputum eosinophilia in patients with asthma were inferred from Sp-EOS ratios of healthy volunteers. The optimal thresholds of sputum eosinophilia were considered as the mean +1.5 standard deviation (SD) of those cell ratios in case of normal distribution, or a 90 percentile otherwise (Belda, et al., 2000; Takaku et al., 2016; Rossios, et al., 2018; Uchida et al., 2019; Hastie, et al., 2021). Thresholds of neutrophilic predominancy in sputum were considered based on the mean + 1.5SD or a 90 percentile of Sp-NEU ratios in healthy volunteers and adopted values from the previous studies (Moore, et al., 2014; Hinks et al., 2016; Takaku et al., 2016; Fricker et al., 2019; Peters, et al., 2019). Patients with severe asthma were classified into four airway-inflammatory subtypes as the following: the paucigranulocyte subtype defined by low Sp-EOS and low Sp-NEU ratio, the eosinophil-predominant subtype defined by high Sp-EOS and low Sp-NEU ratio, the neutrophil-predominant subtype defined by low Sp-EOS and high Sp-NEU ratio, and the mixed-granulocyte subtype defined by high Sp-EOS and high Sp-NEU ratio. We determined the distribution of these subtypes in the type 2 inflammatory subclasses.

### Data Analysis

The normality of the continuous variables was analyzed using the Kolmogorov–Smirnov test. Continuous variables, which were normally distributed, were analyzed using the Student *t-*tests and analysis of variance (ANOVA), while the others were analyzed using the Mann–Whitney U-test and Kruskal–Wallis tests. Categorical variables were analyzed using χ^2^ or Fisher’s exact tests as appropriate. Values are described as mean ± SD or as median with interquartile ranges (IQR), if not normally distributed.

The diagnostic accuracy of the FeNO and B-EOS counts to detect the optimal cutoff value for Sp-EOSs ≥2.7% was evaluated using the receiver operating characteristic curve (ROC) analysis for each variable. An ROC area under the curve of 0.5 was designated as an inability of the biomarker to predict a patient’s correct classification. According to the derived cutoff values, the sensitivity and specificity were calculated.

The time for the first AE after the entry for 12 months and its hazard ratio (HR) were examined for the respective type 2 inflammatory subclasses and the associated use of three biologics, omalizumab and the anti-IL-5 biologics (combination of mepolizumab and benralizumab), using the Kaplan–Meier method and the Cox proportional hazard model. The data for the patients who experienced AEs or were lost to follow-up were censored for the analysis at the times when they occurred. The generalized Wilcoxon signed-rank test and log-rank test were used to assess the differences between the FeNO/B-EOS subgroups and the subgroups with or without biologics in the time to the first AE after the entry. Differences in the unadjusted HRs for the type 2 inflammatory subclasses and the associated biologics used were examined using the Cox proportional hazard model. Before the unadjusted HRs were adjusted, potential explanatory variables were identified using ANOVA, Kruskal–Wallis test, Fisher’s exact test or univariable, Cox proportional hazard model among the type 2 inflammatory subclasses, and the three biologics. Variables with a *p* value < 0.1 at a univariate analysis were entered in the multivariate Cox proportional hazard model as covariates. The data were analyzed using the SPSS version 26.0 (IBM, Armonk, New York, United States).

## Results

A total of 25 healthy volunteers were enrolled but eight of them were excluded; one had severe allergic rhinitis, one had a history of smoking, one exhibited a high FeNO and airway hyperresponsiveness, and five had inadequate sputum samples. Of the 150 patients with severe asthma enrolled, 38 were excluded, because 12 were diagnosed with chronic obstructive pulmonary disease, two were receiving biologics mainly as treatment for eosinophilic rhinosinusitis, and 24 had inadequate sputum samples. In all, 112 patients with severe asthma and 17 volunteers were eligible for the analysis.

The characteristics of the healthy volunteers and the eligible patients with severe asthma at the entry are shown in Table 1. The age and proportion of females were significantly higher among the patients with severe asthma than the healthy volunteers. A total of 69 (61.6%) patients had allergic comorbidities (allergic rhinitis, chronic sinusitis, atopic dermatitis, and urticaria). Most patients took high-dose inhaled corticosteroids (ICS) and a combination with LABA, LTRA, or slow-sustained theophylline, while one third of those received OCS and long-acting muscarinic antagonist (LAMA). Approximately 10% of those previously took biologics. The proportions of eosinophils in sputum and B-EOS counts were significantly higher in patients with severe asthma, while those with neutrophils in sputum were not, compared with those were healthy. The total IgE, FeNO, and some pulmonary function indices were significantly different in the patients with severe asthma compared to those in healthy volunteers.

**TABLE 1:** Demographic and clinical characteristics of the patients at entry.

	Healthy control subject	Patients with severe asthma	*p* value
N	17	112	
Age, years	37.0 (32.0–49.5)	63.0 (53.0–73.0)	**<0.0001**
Female/male, n (%)	5 (29.4)/12 (70.6)	63 (56.2)/49 (43.8)	**0.03**
BMI, kg/m^2^	22.4 (20.4–23.3)	23.2 (21.3–25.3)	NS
Smoking history, no/past/present, n (%)	10 (59.0)	68 (60.7)	NS
	/6 (35.0)	/37 (33.1)	
	/1 (6.0)	/7 (6.2)	
Duration of asthma (y)	NA	14 (6–24)	NA
Allergic rhinitis, n (%)	4 (24)	59 (52.6)	**0.02**
Chronic sinusitis, n (%)	0 (0)	25 (22.3)	**0.03**
Atopic dermatitis, n (%)	0 (0)	12 (10.7)	NS
Urticaria, n (%)	0 (0)	5 (4.4)	NS
LABA/LAMA/LTRA/theophylline, n (%)	NA	108 (96.4)	NA
		/40 (35.7)	
		/99 (88.3)	
		/78 (69.6)	
ICS dose (μg/d)	NA	1,000 (800–1,000)	NA
Oral corticosteroids, n (%)	NA	36 (32.1)	NA
Oral corticosteroids, mg/day	NA	0 (0–4)	NA
Biological treatment before the entry, n (%)	NA	9 (8.0)	NA
Log total IgE, IU/L	1.76 (1.3–2.1)	2.3 (2.0–2.8)	**0.0009**
Blood eosinophil count, /μL	106 (52–177)	271 (89–585)	**0.01**
FeNO, ppb	13 (13–25)	25 (14–85)	**0.01**
Sputum eosinophil ratio, %	0.9 (0.2–2.0)	2.2 (0.4–16.6)	**0.02**
Sputum neutrophil ratio, %	47.5 (29.2–60.3)	40.1 (26.1–57.1)	NS
FEV_1_, % of predicted	94.8 (14.3)	82.2 (20.5)	**0.01**
FVC, % of predicted	99.0 (15.1)	82.3 (20.5)	NS
FEV_1_/FVC, %	82.3 (4.8)	68.3 (14.1)	**<0.0001**

Significant *p* values are shown in bold. The parametric data are expressed as mean (SD); nonparametric data are expressed as median (25%–75%). The Student *t-*test and the Mann–Whitney U-test were performed for parametric continuous variables and nonparametric variables, respectively. Categorical variables were tested by the χ^2^ test or Fisher’s exact test, as appropriate.

ICS, inhaled corticosteroid (fluticasone propriate); 2 μg beclomethasone = 2 μg budesonide = 1 μg fluticasone propriate; 100 μg fluticasone furoate = 500 μg fluticasone propriate.

LABA, long-acting b2-agonist; LAMA, long-acting muscarinic antagonist; LTRA, leukotriene receptor antagonist; NA, not applicable; FeNO, fractional exhaled nitric oxide; y, years.

A total of 75 induced sputum samples (66.7%) were successfully obtained from the patients with severe asthma. The median squamous cell ratios in the sputum samples from healthy volunteers and asthmatics were 6.5% (1.2%–15.2%) and 20.1% (6.6%–40.9%), respectively. As the 90th percentile of the Sp-EOSs for the healthy volunteers, 2.7% was considered as the appropriate cutoff value to distinguish the patients with severe asthma having sputum eosinophilia from those who were not (Supplementary Figure S1). Based on this, we adopted 2.7% as the cutoff value for detecting the patients with sputum eosinophilia. Those who had more than or equal to this value accounted for 48.0% of asthmatics in this study. Similarly, we used 50.0% as the cutoff value to detect the patients with predominant neutrophils in their sputum. This cutoff value for the detection of the predominant Sp-NEUs was considered appropriate in this study since we did not aim to detect the neutrophilic airway diseases, such as chronic bronchitis, and the SARP-II and III study had adopted this cutoff value (Moore, et al., 2014; Peters, et al., 2019). This range has accounted for 34.7% of the patients.

### The Appropriate Cutoff Values of B-EOS Counts and FeNO Levels to Predict Patients With Sputum Eosinophilia

The ROC curve analysis determined that the B-EOS of ≥265 cells/µL and FeNO levels ≥27 ppb optimally distinguished the patients with severe asthma having sputum eosinophilia (>2.7%) from those who were not. The diagnostic accuracies of the cutoff values of B-EOS and FeNO levels were 73.4% [*p* < 0.0001, 95% confidence interval (CI): 61.9–84.9] and 74.8% (*p* < 0.0001, 95% CI: 63.3–86.3), respectively (Supplementary Figure S2). Sensitivity and specificity of the B-EOS cutoff values were 70.3% and 62.5%, respectively, and those of the FeNO were 70.3% and 70.0%, respectively. We adopted these cutoff values for all the patients because these parameters resulted in plausible diagnostic performance.

### Clinical Characteristics of the Four Subgroups Classified by FeNO and B-EOS Cutoff Values

All the patients with severe asthma were allocated into the following four subgroups by the B-EOS and the FeNO cutoff value: 29 in the FeNO^lo^/B-EOS^lo^ subgroup, 24 in the FeNO^hi^/B-EOS^lo^ subgroup, 28 in the FeNO^lo^/B-EOS^hi^ subgroup, and 31 in the FeNO^hi^/B-EOS^hi^ subgroup at the entry (Table 2). The prevalence of four FeNO/B-EOS subgroups was comparable as it ranged from 21.4% to 27.8%. The following indices were not different among the four subgroups: age, sex, body mass index, smoking history, duration of asthma, and prevalence of allergic comorbidities. The proportion of OCS use at the entry was significantly the largest (approximately 50%) in the FeNO^lo^/B-EOS^lo^ subgroup, the second highest (approximately 40%) in the FeNO^hi^/B-EOS^lo^ subgroup, and approximately 20% in the FeNO^hi^/B-EOS^hi^ and FeNO^lo^/B-EOS^hi^ subgroups. The doses of OCS and ICS at the entry did not differ among the four FeNO/B-EOS subgroups (Table 2). The proportions of LABA, LAMA, LTRA, and theophylline use at the entry were also comparable among the four FeNO/B-EOS groups. The prevalence of use of any biologics before the entry was high in the FeNO^hi^/B-EOS^lo^ (about 20%) compared with the other FeNO/B-EOS subgroups. Serum total IgE was the highest in the FeNO^hi^/B-EOS^lo^ subgroup and was significantly different compared with the FeNO^lo^/B-EOS^lo^ subgroup, while the second highest in the FeNO^hi^/B-EOS^hi^ subgroup with significance, compared with that in the B-EOS^lo^ subgroups. The ratio of Sp-EOS was significantly the highest in the FeNO^hi^/B-EOS^hi^ subgroup and the lowest in the FeNO^lo^/B-EOS^lo^ subgroup. The ratios of Sp-NEU were comparable (around 40%) among the subgroups. Most of the pulmonary function parameters were more than 80%, but the FEV_1_/FVC ratios were less than 70% in all the subgroups except FeNO^lo^/B-EOS^lo^. Previous annual AEs were significantly the highest in the FeNO^hi^/B-EOS^lo^ subgroup.

**TABLE 2:** Demographics and clinical characteristics of patients with asthma in the subgroups classified by type 2 biomarker at entry.

	FeNO^lo^	FeNO^hi^	FeNO^lo^	FeNO^hi^	*p* value
	B-EOS^lo^	B-EOS^lo^	B-EOS^hi^	B-EOS^hi^	
N (%)	29 (25.8)	24 (21.4)	28 (25.0)	31 (27.8)	
Age, years	64.0 (47.0–72.0)	58.5 (50.5–73.0)	63.0 (55.7–73.5)	66.0 (54.0–73.0)	NS
Female/male, n (%)	22 (75.8)/7 (24.2)	8 (33.3)/16 (66.7)	15 (53.5)/13 (46.4)	18 (58.0)/13 (42.0)	NS
BMI, kg/m^2^	23.4 (20.5–24.5)	24.2 (21.7–27.1)	23.3 (21.9–24.8)	22.3 (20.1–24.2)	NS
Smoking history, no/past/present, n (%)	20 (69.0)/9 (31.0)/0 (0)	13 (54.1)/9 (37.6)/2 (8.3)	16 (57.1)/10 (35.8)/2 (7.1)	19 (61.2)/9 (29.2)/3 (9.6)	NS
Duration of asthma (y)	10 (5–26)	15 (4–30)	14 (8–18)	14 (4–24)	NS
Allergic rhinitis, n (%)	15 (51.7)	15 (62.5)	15 (53.5)	14 (45.1)	NS
Chronic sinusitis, n (%)	3 (10.3)	4 (16.7)	8 (28.5)	10 (32.2)	NS
Atopic dermatitis, n (%)	4 (13.7)	2 (8.3)	3 (10.7)	3 (9.6)	NS
Urticaria, n (%)	2 (6.8)	1 (4.2)	1 (3.5)	1 (3.2)	NS
Previous annual number of asthma exacerbations	0 (0–5)	3 (1–6) *,†	1 (0–2)	1 (0–4)	**0.04**
LABA/LAMA/LTRA/theophylline, n (%)	26 (89.6)/8 (27.5)	24 (100)/12 (50.0)	28 (100)/6 (21.4)	30 (96.7)/14 (45.1)	NS
	25 (86.2)/19 (65.5)	21 (87.5)/18 (75.0)	26 (92.8)/18 (64.2)	27 (87.1)/23 (74.1)	
ICS dose (μg/d)	1,000 (800–1,000)	1,000 (800–1,000)	1,000 (800–1,000)	900 (800–1,000)	NS
Oral corticosteroids, n (%)	15 (51.7)**	9 (37.5)	5 (17.8)	7 (22.5)	**0.02**
Oral corticosteroids, mg/day	1.0 (0–20)	0 (0–20)	0 (0–5)	0 (0–20)	NS
Biologics before entry, n (%)	3 (3.4)	5 (20.9)	0 (0)	1 (3.2)	NS
Omalizumab/mepolizumab/benralizumab before entry, n (%)	1 (3.4)/1 (3.4)/1 (3.4)	1 (4.1)/2 (8.3)/2 (8.3)	0 (0)/0 (0)/0 (0)	1 (3.2)/0 (0)/0 (0)	NS
Log total IgE, IU/L	2.0 (1.8–2.4)	2.6 (2.1–3.2)^‡^	2.3 (1.8–2.7)	2.4 (2.1–2.9) ^‡‡,††^	**0.006**
Blood eosinophil count, /μL	79 (36–174)	113 (61–187) ^‡‡‡^	484 (331–692) ^‡‡‡,§^	662 (487–1,061) ^‡‡‡^	**<0.0001**
FeNO, ppb	11 (6–16)	88 (42–131) ^‡‡‡,†††^	18 (11–21)	86 (41–121) ^‡‡‡,†††^	**<0.0001**
Sputum eosinophil ratio, %	0.2 (0–1.4)	4.6 (1.7–11.0)	5.3 (0.3–23.8) ^‡‡‡^	14.3 (1.9–29.7) ^‡‡‡/§§^	**<0.0001**
Sputum neutrophil ratio, %	53.8 (34.2–65.9)	36.3 (25.6–67.4)	39.1 (18.7–51.2)	35.1 (25.5–51.0)	NS
FEV_1,_ % of predicted	85.5 (18.7)	82.9 (20.6)	80.0 (24.1)	81.0 (19.7)	NS
FVC, % of predicted	95.2 (18.9)	92.3 (15.8)	85.0 (25.9)	89.2 (18.1)	NS
FEV_1_/FVC, %	72.7 (17.6)	66.8 (11.7)	69.1 (13.7)	65.1 (12.2)	NS

Significant *p* values are shown in bold. The parametric data are expressed as mean (SD); nonparametric data are expressed as median (25%–75%). Oral corticosteroid doses are expressed as median (min–max). ANOVA and the Kruskal–Wallis test were performed for parametric continuous variables and nonparametric variables, respectively. Categorical variables were tested by the χ^2^ test or Fisher’s exact test as appropriate. Further exploration of the results with significant differences in the initial analyses was performed by post hoc pairwise analyses with Bonferroni correction or by the Mann–Whitney U-test as appropriate.

FeNO^lo^, low FeNO; FeNO^hi^, high FeNO; B-EOS^lo^, low B-EOS; B-EOS^hi^, high B-EOS.

ICS, inhaled corticosteroid (fluticasone propriate); 2 μg beclomethasone = 2 μg budesonide = 1 μg fluticasone propriate; 100 μg fluticasone furoate = 500 μg fluticasone propriate.

LABA, long-acting b2-agonist; LAMA, long-acting muscarinic antagonist; LTRA, leukotriene receptor antagonist; NA, not applicable.

*p = 0.04, **p = 0.01; FeNO^hi^/B-EOS^hi^.

†*p* = 0.01, ††*p* = 0.03, †††*p* < .0001; vs. FeNO^lo^/B-EOS^hi^.

‡*p* = 0.01, ‡‡*p* = 0.005, ‡‡‡*p* < .0001; vs. FeNO^lo^/B-EOS^lo^.

§*p* < .0001, §§*p* = 0.03; vs. FeNO^hi^/B-EOS^lo^.

Characteristics of patients who were not treated with any biologics before the entry are shown in Supplementary Table S1. Serum total IgE was significantly higher in the FeNO^hi^/B-EOS^lo^ subgroup than in the B-EOS^hi^ subgroup. The ratio of Sp-EOS gradually increased in the order of FeNO^lo^/B-EOS^lo^, then FeNO^hi^/B-EOS^lo^, then FeNO^lo^/B-EOS^lo^, and then FeNO^hi^/B-EOS^hi^, indicating that the Sp-EOS ratio was significantly lower in the FeNO^lo^/B-EOS^lo^ subgroup than in the other FeNO/B-EOS subgroups.

### Therapeutic Peculiarity of the Four Subgroups for FeNO and B-EOS Cutoff Values

**FIGURE 1:**
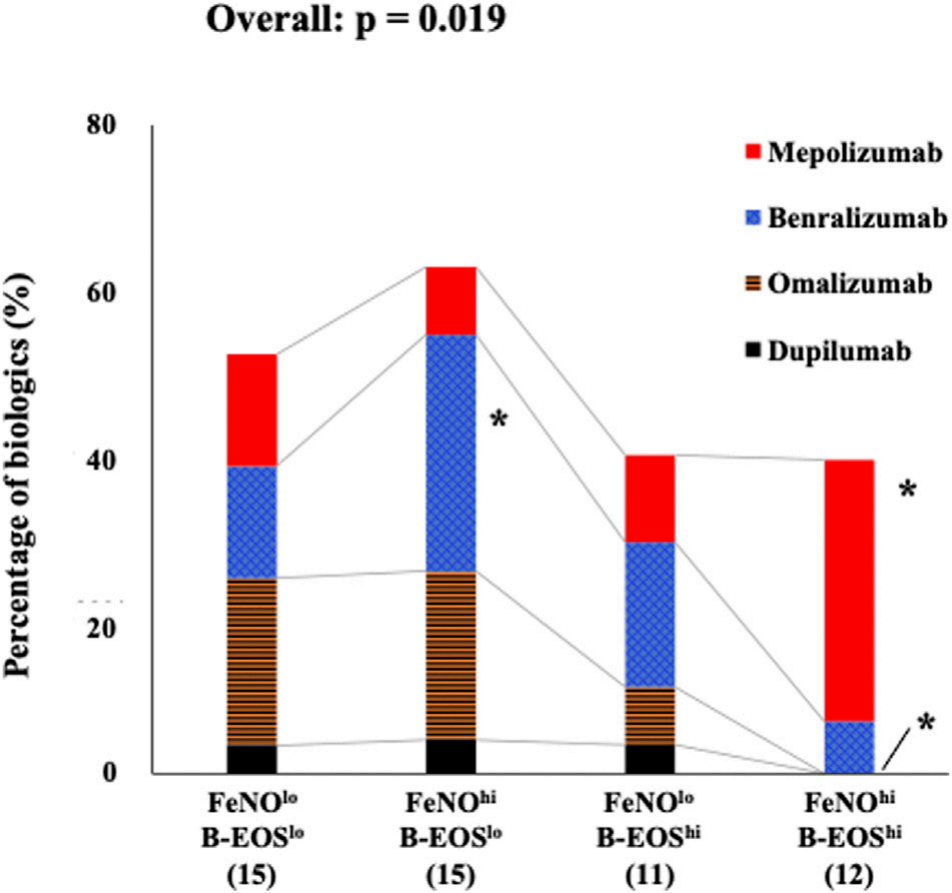
Distribution of the prevalence of biologics use in the type 2 biomarker-classified subgroups. In the low-FeNO/low-B-EOS subgroup, omalizumab, mepolizumab, benralizumab, and dupilumab were as follows: 20.7, 13.8, 13.8, and 3.4%, respectively. In the high-FeNO/low-B-EOS subgroup: 20.8, 8.3, 29.2, and 4.2%, respectively. In the low-FeNO/high-B-EOS subgroup: 7.1, 10.7, 17.9, and 3.6%, respectively. In the high-FeNO/high-B-EOS subgroup: 0.0, 32.3, 6.5, and 0.0%, respectively. Values were analyzed using Fisher’s exact test. *, significantly high or low, compared with the other FeNO/B-EOS subgroups.

The prevalence of any biologic use after the entry was approximately 50% as follows: 16 (55.1%) in the FeNO^lo^/B-EOS^lo^ subgroup, 15 (62.5%) in the FeNO^hi^/B-EOS^lo^ subgroup, 11 (39.2%) in the FeNO^lo^/B-EOS^hi^ subgroup, and 12 (38.7%) in the FeNO^hi^/B-EOS^hi^ subgroup. Distribution of the proportion of biologics use after the entry were significantly different among the FeNO/B-EOS subgroups (*p* = 0.019). The proportion of benralizumab use was significantly higher in the FeNO^hi^/B-EOS^lo^ subgroup, while mepolizumab use was significantly higher in the FeNO^hi^/B-EOS^hi^ subgroup (Figure 1). The proportion of omalizumab was significantly lower in the FeNO^hi^/B-EOS^hi^ subgroup, while it was almost equal in the other subgroups. The proportion of dupilumab and no biologic use was comparable between the FeNO/B-EOS subgroups.

### Comparison of Risk of Future AEs Among the Four Subgroups Classified Based on FeNO and the B-EOS Cutoff Values

**FIGURE 2:**
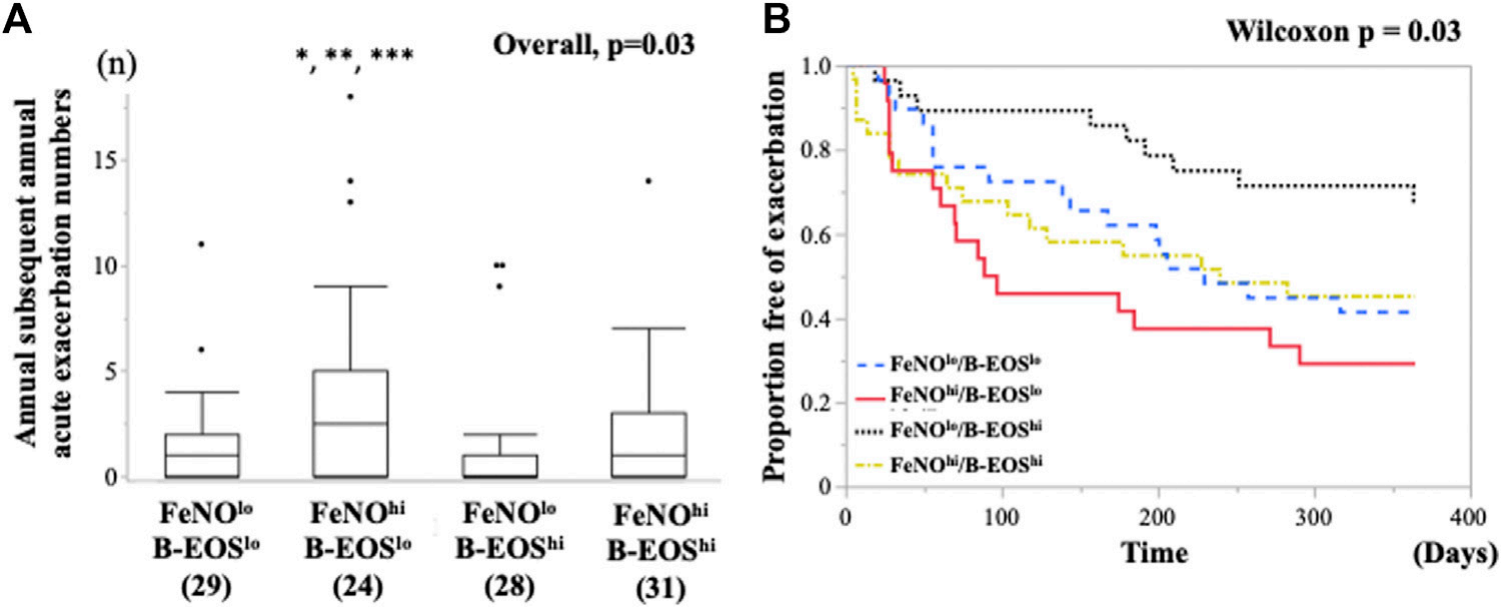
Comparison of the future risk of asthma exacerbations after the entry between the type 2 biomarker-classified subgroups. **(A)** Differences in the number of annual subsequent asthma exacerbation after the entry among the subgroups classified by FeNO and B-EOS cutoff values for detection of sputum eosinophilia. Horizontal bars represent median. *vs. Low FeNO, High-b-EOS, *p* = 0.006; **vs. Low FeNO, Low-b-EOS, *p* = 0.01; ***vs. High FeNO, High-b-EOS, *p* = 0.04. **(B)** Kaplan–Meier analysis for asthma exacerbation-free time after the entry among the predominant type 2 inflammation subgroups. Generalized Wilcoxon, *p* = 0.048, Log Rank, *p* = 0.07. B-EOS, blood eosinophils; AE, asthma exacerbation.

The numbers of the annual AEs after the entry were significantly different among the FeNO/B-EOS subgroups (Figure 2A), which was highest in the FeNO^hi^/B-EOS^lo^ [median 3 (0–5)] among the FeNO/B-EOS groups. Subsequent annual AE numbers were lowest in the FeNO^lo^/B-EOS^hi^ [median 0 (0–1)] and those in the FeNO^hi^/B-EOS^hi^ [median 1 (1–3)] were mostly equal in the FeNO^lo^/B-EOS^lo^ groups [median 1 (0–2)].

The subsequent AE-free time for 12 months after the entry was significantly different among the FeNO/B-EOS subgroups, which was assessed using the Kaplan–Meier-Meier method (Generalized Wilcoxon, *p* = 0.048, Log Rank, *p* = 0.07) (Figure 2B). The median AE-free times were 230 days (139, 95%CI) in the FeNO^lo^/B-EOS^lo^ subgroup, 93 days (56, 95%CI) in the FeNO^hi^/B-EOS^lo^, 365 days (364, 95%CI) in the FeNO^lo^/B-EOS^hi^, and 240 days (75, 95%CI) in the FeNO^hi^/B-EOS^hi^. The unadjusted HR for the subsequent AE-free time after the entry was significantly higher in the FeNO^hi^/B-EOS^lo^ subgroup, compared with the FeNO^lo^/B-EOS^hi^, based on the univariable Cox proportional hazard model (Table 3). The unadjusted HRs for the subsequent AE-free time of age, the number of previous AEs over 12 months, and the use of omalizumab were also significant. Aging was associated with the longer subsequent AE-free time after the entry. An increasing number of previous AEs for 12 months and omalizumab use was associated with the shorter subsequent AE-free time after the entry. Three patients who received dupilumab were excluded from the former analysis. There were no significances in the following unadjusted HRs of potent risk factors for the annual subsequent AEs: sex, chronic sinusitis, use of OCS, use of biologics, %FEV1, and FEV/FVC (Table 3). The HR of the patients in the FeNO^hi^/B-EOS^lo^ subgroup did not persist significantly after adjustment with the confounding variables using the multivariable Cox proportional hazard model (Table 3). The adjusted HR for age and the previous annual AE numbers that persisted independently were significant.

**TABLE 3:** Univariable and multivariable Cox proportional hazard models to determine the hazard ratio for the subsequent asthma exacerbation-free time in the FeNO/B-EOS subgroups.

Univariate analysis			Multivariate analysis	
Factor	Unadjusted HR (95%CI)	*p* value	Adjusted HR (95%CI)	*p* value
Aging	0.98 (0.96–0.99)	**0.018**	0.98 (0.96–0.99)	**0.04**
Sex	1.00 (reference)	**-**		
Male	1.10 (0.67–1.81)	N.S.		
Female				
Chronic sinusitis	1.00 (reference)	**-**		
No	0.97 (0.53–1.79)	N.S.		
Yes				
Oral corticosteroids	1.00 (reference)	**-**		
No	1.18 (0.70–1.98)	N.S.		
Yes				
Biologics after the entry	1.00 (reference)	**-**	1.00 (reference)	**-**
Omalizumab	0.49 (0.23–1.03)	**0.059**	0.58 (0.27–1.26)	N.S.
Anti-IL-5 antibody	0.53 (0.27–1.06)	N.S.	1.16 (0.57–2.50)	N.S.
No biologics				
Total IgE	1.00 (reference)	**-**		
No	1.00 (1.00–1.00)	N.S.		
Yes				
FEV_1**,**_ % of predicted	1.00 (0.98–1.02)	N.S.		
FEV_1_/FVC**,** %	1.00 (0.98–1.02)	N.S.		
Numbers of annual previous AEs	1.13 (1.09–1.18)	**<0.0001**	1.15 (1.09–1.18)	**<0.0001**
FeNO/B-EOS subgroups	1.00 (reference)	**-**	1.00 (reference)	**-**
FeNO^hi^/B-EOS^lo^	0.33 (0.15–0.70)	**0.004**	0.57 (0.25–1.31)	N.S.
FeNO^lo^/B-EOS^hi^	0.66 (0.34–1.28)	N.S.	0.66 (0.33–1.30)	N.S.
FeNO^lo^/B-EOS^lo^	0.61 (0.31–1.21)	N.S.	1.02 (0.47–2.21)	N.S.
FeNO^hi^/B-EOS^hi^				

Cox proportional hazard model was used for this analysis.

FeNO^lo^, low FeNO; FeNO^hi^, high FeNO; B-EOS^lo^, low B-EOS; B-EOS^hi^, high B-EOS.

The bold values means statisticaly significant.

We examined this HR for the FeNO^hi^/B-EOS^lo^ subgroup with adjusted biologics therapies including omalizumab, anti-IL-5 biologics (mepolizumab + benralizumab), and non-biologics. The HR of the patients in the FeNO^hi^/B-EOS^lo^ subgroup persisted significantly higher than the FeNO^lo^/B-EOS^hi^ subgroup (*p* = 0.008) (Table 4).

**TABLE 4:** Adjusted hazard ratio for the subsequent asthma exacerbation-free time in the FeNO/B-EOS subgroups with biologics use as the confounding variable.

Factor	Adjusted HR (95%CI)	*p* value
Biologics after the entry	1.00 (reference)	**-**
Omalizumab	0.53 (0.25–1.15)	N.S.
Anti-IL-5 antibody	0.64 (0.31–1.31)	N.S.
No biologics		
FeNO/B-EOS subgroups	1.00 (reference)	**-**
FeNO^hi^/B-EOS^lo^	0.35 (0.16–0.76)	**0.008**
FeNO^lo^/B-EOS^hi^	0.66 (0.34–1.29)	N.S.
FeNO^lo^/B-EOS^lo^	0.68 (0.33–1.40)	N.S.
FeNO^hi^/B-EOS^hi^		

Cox proportional hazard model was used for this analysis.

FeNO^lo^, low FeNO; FeNO^hi^, high FeNO; B-EOS^lo^, low B-EOS; B-EOS^hi^, high B-EOS.

The bold values means statisticaly significant.

### Distribution of Sputum Granulocytic Subtypes in the FeNO/B-EOS Subgroups

**FIGURE 3:**
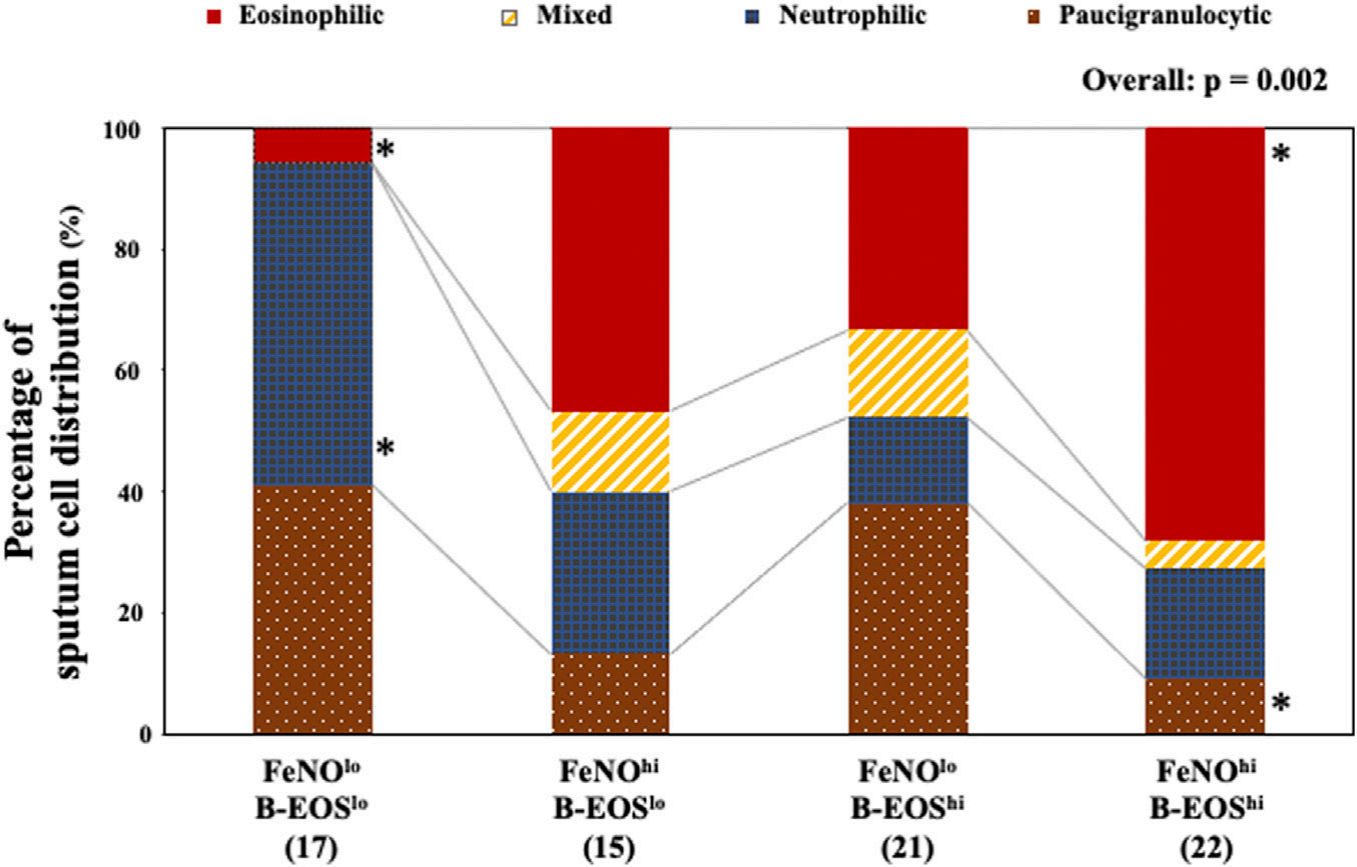
Distribution of sputum granulocyte-predominant subtypes in the type 2 biomarker-classified subgroups. Of successful sputum induction, 75 adequate sputum samples were examined. Patients were divided into four subtypes according to the cutoff values of sputum eosinophilia (≥2.7%) and predominant neutrophil (≥50.0%). Values were analyzed using Fisher’s exact test. In the FeNO^lo^/B-EOS^lo^ subgroup, the eosinophil-predominant, mixed-granulocyte, neutrophil-predominant, and paucigranulocyte subtypes were as follows: 5.9, 0.0, 52.9, and 41.2%, respectively. In the FeNO^hi^/B-EOS^lo^ subgroup: 46.7, 13.3, 26.7, and 13.3%, respectively. In the FeNO^lo^/B-EOS^hi^ subgroup: 33.3, 14.3, 14.3, and 38.1%, respectively. In the FeNO^hi^/B-EOS^hi^ subgroup: 68.2, 4.5, 18.2, and 9.1%, respectively. FeNO^lo^, low FeNO; FeNO^hi^, high FeNO; B-EOS^lo^, low B-EOS; B-EOS^hi^, high B-EOS. *, significantly high or low, compared with the other FeNO/B-EOS subgroups.

The FeNO/B-EOS subgroups exhibited heterogeneous distribution of the four subtypes with predominant granulocytes in the sputum (Figure 3). The FeNO^hi^/B-EOS^hi^ subclass exhibited the largest significant population of the sputum eosinophil-predominant subtype (68.2%), the smallest significant population of the sputum paucigranulocyte subtype (9.1%), and marginal populations of the sputum mixed-granulocytic subtype (4.5%) and the sputum neutrophil-predominant subtype (18.2%), compared with the other FeNO/B-EOS subgroups. The FeNO^lo^/B-EOS^lo^ subgroup exhibited the smallest significant population of the eosinophil-predominant subtype (5.9%), the largest significant population of the neutrophil-predominant subtype (52.9%), the largest population of the paucigranulocyte subtype (41.2%), and the smallest population of the mixed-granulocyte subtype (0.0%). The FeNO^hi^/B-EOS^lo^ subgroup exhibited the second largest population of the eosinophil-predominant subtype (46.7%), the second largest population of the neutrophil-dominant subtype (26.7%), and moderate populations of the mixed-granulocyte (13.3%) and paucigranulocyte subtypes (13.3%). The FeNO^lo^/B-EOS^hi^ subgroup exhibited the third largest population of the eosinophil-predominant subtype (33.3%), the second largest significant population of the paucigranulocyte subtype (38.1%), the smallest significant population of the neutrophil-dominant subtype (14.3%), and moderate population of the mixed-granulocytic subtype (14.3%).

The classification into four subtypes by predominant sputum granulocytes did not show an effect on HR for subsequent AEs-free time and reduction of that with anti-IL-5 biologics use as shown by the multivariable Cox proportional hazard model (data not shown).

### Comparison of Improvement Efficacy for Future AEs Between the Patients Treated With Omalizumab and Anti-IL-5 Biologics

Since the use of biologics depended on the FeNO/B-EOS subgroups, we examined the effect of biologics on the occurrence of the first AE for a year after the entry day (as the day of any biologics beginning). Patients treated with dupilumab were excluded from this analysis because there were only a few of them in this study. The characteristics of the patients are shown in Table 5. OCS use was significantly higher in the biologics group, especially in the omalizumab group, compared with the nonbiologics group. The total IgE was significantly higher in the anti-IL-5 biologics and nonbiologics groups, compared with the omalizumab group. B-EOS was significantly higher in the anti-IL-5 biologics and nonbiologics groups, compared with the omalizumab group. The previous annual AEs were significantly higher in the omalizumab group. There were no differences in the other indices.

**TABLE 5:** Demographics and clinical characteristics of patients with asthma in groups of biologic drugs.

	No biologics	Omalizumab	Anti-IL-5 biologics*	*p* value
N (%)	59 (54.1)	13 (11.9)	37 (34.0)	
Age, years	63.0 (51.0–72.0)	55.0 (46.5–65.0)	70.0 (59.5–74.5)	NS
Female/male, n (%)	25 (42.3)/34 (57.6)	8 (61.5)/5 (38.4)	28 (77.7)/8 (22.3)	NS
BMI, kg/m^2^	23.3 (21.5–25.5)	24.2 (21.2–25.5)	23.0 (20.3–24.2)	NS
Chronic sinusitis, n (%)	13 (22.0)	0 (0)	10 (27.7)	NS
ICS dose (μg/d)	1,000 (800–1,000)	1,000 (800–1,350)	1,000 (800–1,000)	NS
Oral corticosteroids, n (%)	7 (11.8)	8 (61.5)†	18 (48.6)†	**<0.0001**
Log total IgE, IU/L	2.3 (2.1–2.9)‡	1.8 (1.2–2.3)	2.2 (2.0–2.6)‡‡	**0.006**
Blood eosinophil counts ,/μL	328 (179–559)‡‡‡	121 (24–225)	428 (70–724)‡‡‡‡	**0.0083**
FeNO, ppb	25 (11–84)	18 (10–47)	36 (17–111)	NS
Sputum eosinophil ratio**,** % §	2.1 (0.4–16.5)	0.5 (0.1–5.9)	9.1 (0.3–40.5)	NS
Sputum neutrophil ratio**,** % §	40.6 (23.9–56.7)	63.7 (44.3–77.4)	34.1 (24.0–56.0)	NS
Deviation of sputum leukocyte subtypes**,** n (%)	23 (41.0)/14 (25.0)/3 (5.3)/16 (28.5)	0 (0)/2 (50)/1 (25) 1 (25)	6 (50.0)/3 (25.0)/2 (16.6)/1 (8.3)	NS
Eosinophilic**/**neutrophilic**/**mixed**/**paucigranulocytic				
FEV_1,_ % of predicted	79.6 (21.2)	91.1 (18.4)	83.1 (20.4)	NS
FEV_1_/FVC**,** %	68.8 (13.8)	73.0 (17.9)	64.8 (13.3)	NS
Previous annual number of asthma exacerbations, (%)	1 (0–2)	5 (1–8)†††	3 (1–6)††††	**<0.0001**

Significant *p* values are shown in bold. The parametric data are expressed as mean (SD); nonparametric data are expressed as median (25%–75%). ANOVA and the Kruskal–Wallis test were performed for parametric continuous variables and nonparametric variables, respectively. Categorical variables were tested by the χ^2^ test or Fisher’s exact test as appropriate. Further exploration of the results with significant differences in the initial analyses was performed by post hoc pairwise analyses with Bonferroni correction or by the Mann–Whitney U-test as appropriate.

*, anti-IL-5 biologics, mepolizumab + benralizumab.

§, sputum neutrophil and eosinophil ratio; no biologics n = 56, omalizumab n = 4, and anti-IL-5 biologics *n* = 12.

Seventy-five sputum samples are obtained.

†*p* < 0.0001, ††*p* < 0.0001, †††*p* = 0.0001, ††††*p* = 0.0006; vs. no biologics.

‡*p* = 0.001, ‡‡*p* = 0.03, ‡‡‡*p* = 0.0007, ‡‡‡‡*p* = 0.04; vs. omalizumab.

**FIGURE 4:**
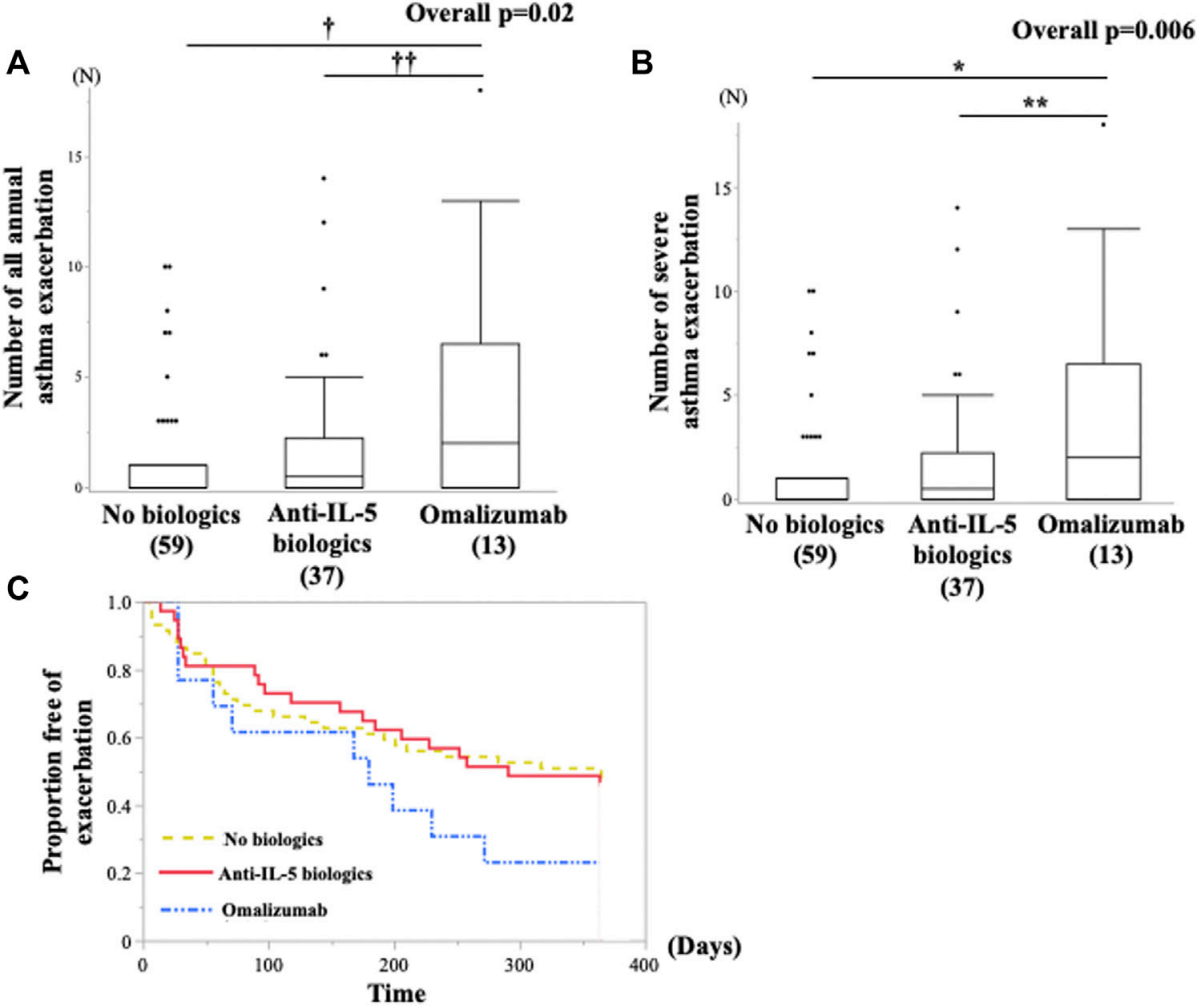
Comparison of the future risk of asthma exacerbations after the entry between the groups of biologics use annual subsequent **(A)** all asthma exacerbations and **(B)** severe asthma exacerbations was examined using the Kruskal–Wallis test and Bonferroni correction for post hoc pairwise analyses. **(C)** All asthma exacerbations-free time from the entry are examined using Kaplan–Meier analysis. (A)*vs. no biologics, *p* = 0.001; **vs. mepolizumab and benralizumab, *p* = 0.01. **(B)**†vs. no biologics, *p* = 0.0007; ††vs. mepolizumab and benralizumab, *p* = 0.01.

The subsequent annual AEs and SAEs after the entry was significantly higher in the omalizumab group than those in the anti-IL-5 biologics and nonbiologics groups (Figures 4A,B). We also examined whether the effect of omalizumab and anti-IL-5 biologics on subsequent annual AEs and SAEs after the entry differed among the FeNO/B-EOS subgroups. All patients receiving omalizumab or anti-IL-5 biologics in the FeNO^hi^/B-EOS^lo^ subgroup frequently experienced AEs and SAEs without significance, compared with the other FeNO/B-EOS subgroups (Supplementary Figure S3A). The patients initiated newly on these biologics significantly experienced frequent AEs and SAEs (Supplementary Figure S3B). The Kaplan–Meier method did not show any differences in the AE-free time after the entry among the three treatment groups (Figure 4C).

## Discussion

This study determined that the classification of patients with severe asthma into four subgroups according to FeNO and B-EOS contributed to show differences in frequency of future annual AEs, the heterogeneous prevalence of subclasses stratified by Sp-EOS and Sp-NEU rates, and particular patterns of clinical response to two classes of biologics, namely anti-IgE and anti-IL-5. The use of FeNO and B-EOS as biomarkers provides benefits for the treatment of severe eosinophilic asthma with approved biologics since they are expected to predict the people who respond to biologics (Hanania, et al., 2013; Casale et al., 2019; Ortega, et al., 2016; Harvey, et al., 2020; Kavanagh, et al., 2020; Bleecker, et al., 2020; Kroes et al., 2020; Kavanagh, et al., 2021; Eger, et al., 2021). In this study, the FeNO^hi^/B-EOS^lo^ subgroups but not the FeNO^hi^/B-EOS^hi^ subgroups experienced a high recurrence of future annual AEs and restricted improvement in annual AEs following biologics use, suggesting that a sole increase of FeNO may serve as an independent biomarker for biologics use in difficult-to-treat asthma. Furthermore, in this study, variable proportions of the four subtypes based on the predominant Sp-EOS and Sp-NEU rate indicates the complexity of airway inflammation that underlies the FeNO/B-EOS subgroups. This finding may help in identifying precision medicine strategies. Likewise, specific use of biologics in the subgroups classified by type 2 biomarkers in this study proved that the combination of FeNO and B-EOS may be a feasible strategy for the use of type 2 biomarkers for choice of biologics. Taken together, the characterization of type 2 inflammation-predominant severe asthma based on the classification by two dimensional-type 2 biomarkers in this study might be more convincing.

The patients with severe asthma in the FeNO^hi^/B-EOS^hi^ subgroup exhibited both the highest total IgE and Sp-EOS ratios in this study. Most of those patients also belonged to the eosinophil-predominant subtype in this study. These results suggest that both the Th2 system and cascades to especially induce eosinophilic airway inflammation, including ILC2, might be enhanced in most of the FeNO^hi^/B-EOS^hi^ subgroup. Genomics analysis studies have shown median gene upregulation of IL-4, IL-5, and IL-13 mRNA in patients with severe asthma and who expressed the features of type 2 inflammatory asthma (Peters, et al., 2019). Our findings are in line with this study.

The highest prevalence of patients with severe asthma in the FeNO^hi^/B-EOS^hi^ subgroup received mepolizumab. This result is reasonable because the eosinophil-predominant subtype is prominent in this study. Several studies demonstrated that B-EOS may serve as a predictor of response to mepolizumab (Ortega, et al., 2016; Harvey, et al., 2020; Kavanagh, et al., 2020). A longitudinal study addressed that the responders defined that reduction of ACT scores to mepolizumub included an increase of B-EOS at the baseline (Kavanagh, et al., 2020). In fact, the present study demonstrated that the frequency of the future annual AEs in the FeNO^hi^/B-EOS^hi^ subgroups was not the highest, although the risk of annual AEs was the highest in the previous study (Malinovschi, et al., 2013; Soma, et al., 2018; Busse, et al., 2021). This discrepancy in the present study may partially be due to mepolizumub administration because anti-IL-5 biologics decrease HR for the time to subsequent AE of patients with FeNO^hi^/B-EOS^hi^. Therefore, mepolizumub seems appropriate in this subgroup.

This study demonstrated that the FeNO^hi^/B-EOS^lo^ subgroup had the highest total IgE among the FeNO/B-EOS subgroups. We have previously reported that severe asthma with FeNO^hi^/B-EOS^lo^ exhibited the highest number of sensitized allergens (Soma, et al., 2018). FeNO is generated by the bronchial epithelial cells mainly through an IL-13-driven pathway (Chibana, et al., 2008; Matsunaga et al., 2021). Our results suggest that the type 2 immunologic response, especially related to IL-4/IL-13, is accelerated in them. Additionally, a low level of E-BOS may predict a lower Sp-EOS ratio than high E-BOS in this subgroup (Table 2). Thus, the immunologic pathway might skew to IL-4/IL-13-oriented system and mild local eosinophilic inflammation in this subgroup. In contrast, the prevalence of those with the sputum neutrophil-predominant was relatively high in the FeNO^hi^/B-EOS^lo^ group, compared with the B-EOS^hi^, suggesting that type 2-low airway inflammation coexisted in this subgroup. This complicated underlying immune pathway may be involved in AEs resistant to biologics. Indeed, our findings showed that resident future AEs and HR for the AE-free time were insufficiently modified by biologics, including anti-IgE or anti-IL-5 biologics.

The highest HR of subsequent AE-free time was observed in the FeNO^hi^/B-EOS^lo^ subgroup among the FeNO/B-EOS subgroups, especially compared within the FeNO^lo^/B-EOS^hi^ subgroup in this study. In addition, the previous annual AEs were the most frequent in this subgroup. Though the HR improved after adjustment with biologics use, the adjusted HR in the FeNO^hi^/B-EOS^lo^ persisted the highest, which was significant compared with the FeNO^lo^/B-EOS^hi^. Improvement in the frequency of the subsequent annual AEs and SAEs after the entry with the biologics in the FeNO^hi^/B-EOS^lo^ subgroup was not superior to that in the other FeNO/B-EOS subgroups. These results suggest that the FeNO^hi^/B-EOS^lo^ subgroup exhibited markedly uncontrolled condition and were resistant to biologics. On the contrary, the unadjusted HR for the subsequent AE-free time after the entry day was lower in the B-EOS^hi^ subgroups than in the FeNO^hi^/B-EOS^lo^. The discrepancy between the previous studies (Malinovschi, et al., 2013; Soma, et al., 2018; Busse, et al., 2021) and the present study may be due to the specific biologics used in the B-EOS^hi^ subgroups.

A high proportion of patients with severe asthma in the FeNO^hi^/B-EOS^lo^ subgroup received benralizumub. A longitudinal study showed that the high FeNO at the baseline was a predictor of the super responders to benralizumub (Kavanagh, et al., 2021). A study reported that FeNO ≥40ppb predicted the responders of benralizumub (Watanabe, et al., 2021). Considering the available evidence, FeNO levels may help decide the possible administration of benralizumub. However, the effect of biologics on the AEs was limited in this subgroup. It may be partly due to the complex nature of airway leukocytic inflammation (Figure 3). Taken together, anti-IL-5 biologics may be preferable for the patients with extensively high FeNO in the FeNO^hi/^B-EOS^hi^.

Notably, anti-IL-5 biologics had been administrated to 16.6% of the patients in the FeNO^hi^/B-EOS^lo^ subgroup before the entry. They had abrogated B-EOS counts, possibly resulting in modification of the prevalence of the FeNO/B-EOS subgroups. Alternative biomarkers for B-EOS are needed because prior administration of biologics could change the population of the FeNO/B-EOS subgroup following biologics therapy. A prospective observation study showed that in severe eosinophilic asthma treated with mepolizumab, high FeNO could be used to distinguish residual eosinophilic exacerbations from the noneosinophilic exacerbations (McDowell, et al., 2021). It suggests that FeNO can be a useful predictive marker for the eosinophilic exacerbations following anti-IL-5 antibody therapy.

The FeNO^lo^/B-EOS^hi^ subgroup showed the lowest prevalence of patients with increased sputum granulocytes and half of the patients with predominant eosinophils in sputum (the sputum eosinophil-predominant + the mixed-granulocyte subtype). Anti-IL-5 biologics were mostly administrated in this subgroup, resulting in the lowest future risk of the AEs among the FeNO/B-EOS subgroups. Considering the main target cells of anti-IL-5 biologics, it is understandable how the lower future risk of AEs was possible. Therefore, anti-IL-5 biologics may be preferable for patients with FeNO^lo^/B-EOS^hi^.

In the FeNO^lo^/B-EOS^lo^ subgroup, future annual AEs remained high unless more than half of the patients received any biologics and OCS. The neutrophil-predominant and paucigranulocyte subtypes were the primary subtypes in this subclass, suggesting that the eosinophilic airway inflammation underlying the FeNO^lo^/B-EOS^lo^ was not involved in mitigation of the risk of future AEs. The patients treated with omalizumab experienced significantly more frequent subsequent annual AEs and SAEs in this subgroup (Supplementary Figure S3), suggesting omalizumab might be not suitable in this subgroup. Further studies are required to explore precision therapy needed for this subgroup.

Endotype classification based on predominant inflammatory cells in sputum revealed that the FeNO/B-EOS-classified subgroups comprised of various proportions of granulocytes in the sputum. The complexity of granulocyte-classified subclasses seemed to influence severe asthma control after the initiation of biologics use. Indeed, compared within the FeNO^hi^/B-EOS^lo^, the numbers of the future annual AEs were low in the FeNO^hi^/B-EOS^hi^ where the primary subtype was the eosinophil-predominant one (Figure 2A) and only anti-IL5 biologics were added on. In contrast, the numbers of the future annual AEs were remarkably high in the FeNO^hi^/B-EOS^lo^ subgroup, where the neutrophil-predominant subtype was also predominant, which was at similar levels to the eosinophil-predominant subtype (40%). In addition, Sp-EOS rates were smaller in the FeNO^hi^/B-EOS^lo^ subgroup than in the B-EOS^hi^ subgroups (Table 2). This might lead to an attenuating contribution of biologics to the numbers of future annual AEs. In the FeNO^lo^/B-EOS^hi^ group, the numbers of the future annual AEs were the smallest among the type 2 biomarker subgroups partly because the eosinophil-predominant subtype accounted for almost of patients in this subgroup and granulocytes were low as compared with the others.

We found that asthmatics treated with anti-IL-5 biologics and omalizumab maintained a similar HR of subsequent AE-free time to a magnitude that those who were not treated with them experienced. In this study, the patients treated with biologics seemed more severe than those who were not treated with them since patients who were treated with biologics frequently experienced annual AEs in the previous year and had a high proportion of the OCS use, compared with those who were not treated with biologics. Considering this result, the comparable HRs between the add-on and not-add-on biologics seemed to verify the benefit of biologics for AEs.

Patients with severe asthma treated with omalizumab and anti-IL-5 biologics frequently experienced subsequent annual AEs and SAEs after the entry in the FeNO^hi^/B-EOS^lo^ subgroup, compared with the other FeNO/B-EOS subgroups (Supplementary Figure S3), suggesting that these biologics were partially effective in the FeNO^hi^/B-EOS^lo^ subgroups. This may be a factor in the more frequent annual AEs and SAEs in the FeNO^hi^/B-EOS^lo^ subgroup.

There are several limitations in this study. The analysis of distribution of leukocytes in sputum was only performed in a small number of patients. Induction of sputum is difficult to obtain sufficient volumes and adequate samples in real practice. Our success rate of sputum induction was similar to that of the previous study (Tanaka et al., 2021). This study was of value in clarifying distinctive locations of the subtype based on the rates of sputum leukocytes using scale of sputum samples almost equal to those in the previous studies. Tendency of biologics administration and their effectiveness on AEs were restrictively evaluated. However, the present study is significant regarding that it shows the superior efficacy of anti-IL-5 biologics in preserving AE-free time to omalizumab. A few indirect treatment studies and a Cochrane analysis reviewed controversial results of the effectiveness of mepolizumub, benralizumab, and reslizumab on reduction of the annual rate of SAEs compared with placebo (Farne, et al., 2017; Ramonell and Iftikhar, 2020; Agache et al., 2020; Bousquet, et al., 2021). This study was a retrospective observational study of small scale, and further prospective and large-scale studies are needed.

In conclusion, the present study addresses that the classification according to two representative type 2 biomarkers demonstrating four specific clusters in severe asthma that present distinct patterns of four airway-inflammatory subtypes and a pattern of biologics use in real-world practice. This clustering by type 2 biomarkers seems to be consistently convenient for making decisions on biologics use, and likewise, to include complexity in airway inflammation that might be involved in the resident AE and tolerance to these therapeutic agents. For biologics therapy, this classification suggests that patients with severe asthma who have solely high FeNO may most likely have refractory type 2 severe asthma. The convenient type 2 of the biomarker classification likely offers useful information that would facilitate the decisions on initial biologics use in patients with severe asthma and help decide with a step afterward.

## Data Availability

The original contributions presented in the study are included in the article/Supplementary Material, further inquiries can be directed to the corresponding author.

## Abbreviations

AE, asthma exacerbation; ANOVA, analysis of variance; ATS/ERS, American Thoracic Society/European Respiratory Society; B-EOS, blood eosinophil count; B-EOS^hi^, high B-EOS; B-EOS^lo^, low B-EOS; CI, confidence interval; FeNO, fraction of exhaled nitric oxide; FeNO^hi^, high FeNO; FeNO^lo^, low FeNO; GINA, Global Initiative for Asthma; HR, hazard ratio; ICS, inhaled corticosteroids; IQR, interquartile range; LABA, long-acting β-agonist; LAMA, long-acting muscarinic antagonist; LTRA, leukotriene receptor antagonist; OCS, oral corticosteroid; ppb, part per billion; ROC, receiver operating characteristic curve; SAE, severe asthma exacerbation; SD, standard deviation; Sp-EOS, sputum eosinophil; Sp-NEU, sputum neutrophil.

## References

[B1] Agache I. Beltran J. Akdis C. Akdis M. Canelo-Aybar C. Canonica G. W. (2020). Efficacy and Safety of Treatment with Biologicals (Benralizumab, Dupilumab, Mepolizumab, Omalizumab and Reslizumab) for Severe Eosinophilic Asthma. A Systematic Review for the EAACI Guidelines - Recommendations on the Use of Biologicals in Severe Asthma. Allergy 75 (5), 1023–1042. 10.1111/all.14221 32034960

[B2] American Thoracic SocietyEuropean Respiratory Society (2005). ATS/ERS Recommendations for Standardized Procedures for the Online and Offline Measurement of Exhaled Lower Respiratory Nitric Oxide and Nasal Nitric Oxide, 2005. Am. J. Respir. Crit. Care Med. 171 (8), 912–930. 10.1164/rccm.200406-710ST 15817806

[B3] Belda J. Leigh R. Parameswaran K. O'Byrne P. M. Sears M. R. Hargreave F. E. (2000). Induced Sputum Cell Counts in Healthy Adults. Am. J. Respir. Crit. Care Med. 161 (2), 475–478. 10.1164/ajrccm.161.2.9903097 10673188

[B4] Bleecker E. R. Wechsler M. E. FitzGerald J. M. Menzies-Gow A. Wu Y. Hirsch I. (2020). Baseline Patient Factors Impact on the Clinical Efficacy of Benralizumab for Severe Asthma. Eur. Respir. J. 52 (4), 1800936. 10.1183/13993003.00936-2018 PMC6203407 30139780

[B5] Bousquet J. Humbert M. Gibson P. G. Kostikas K. Jaumont X. Pfister P. (2021). Real-World Effectiveness of Omalizumab in Severe Allergic Asthma: A Meta-Analysis of Observational Studies. J. Allergy Clin. Immunol. Pract. 9 (7), 2702–2714. 10.1016/j.jaip.2021.01.011 33486142

[B6] Buhl R. Bel E. Bourdin A. Dávila I. Douglass J. M. Jackson D. J. (2022). Effective Management of Severe Asthma with Biologic Medications in Adult Patients: A Literature Review and International Expert Opinion. J. Allergy Clin. Immunol. Pract. 10 (2), 422–432. 10.1016/j.jaip.2021.10.059 34763123

[B7] Busse W. W. Wenzel S. E. Casale T. B. FitzGerald J. M. Rice M. S. Daizadeh N. (2021). Baseline FeNO as a Prognostic Biomarker for Subsequent Severe Asthma Exacerbations in Patients with Uncontrolled, Moderate-To-Severe Asthma Receiving Placebo in the LIBERTY ASTHMA QUEST Study: a post-hoc Analysis. Lancet Respir. Med. 9 (10), 1165–1173. 10.1016/S2213-2600(21)00124-7 34181876

[B8] Casale T. B. Luskin A. T. Busse W. Zeiger R. S. Trzaskoma B. Yang M. (2019). Omalizumab Effectiveness by Biomarker Status in Patients with Asthma: Evidence from PROSPERO, A Prospective Real-World Study. J. Allergy Clin. Immunol. Pract. 7 (1), 156–e1. 10.1016/j.jaip.2018.04.043 29800752

[B9] Chibana K. Trudeau J. B. Mustovich A. T. Mustovitch A. T. Hu H. Zhao J. (2008). IL-13 Induced Increases in Nitrite Levels Are Primarily Driven by Increases in Inducible Nitric Oxide Synthase as Compared with Effects on Arginases in Human Primary Bronchial Epithelial Cells. Clin. Exp. Allergy 38 (6), 936–946. 10.1111/j.1365-2222.2008.02969.x 18384429 PMC11934259

[B10] Chung K. F. Wenzel S. E. Brozek J. L. Bush A. Castro M. Sterk P. J. (2014). International ERS/ATS Guidelines on Definition, Evaluation and Treatment of Severe Asthma. Eur. Respir. J. 43 (2), 343–373. 10.1183/09031936.00202013 24337046

[B12] Denlinger L. C. Phillips B. R. Ramratnam S. Ross K. Bhakta N. R. Cardet J. C. (2017). Inflammatory and Comorbid Features of Patients with Severe Asthma and Frequent Exacerbations. Am. J. Respir. Crit. Care Med. 195 (3), 302–313. 10.1164/rccm.201602-0419OC 27556234 PMC5328178

[B14] Dweik R. A. Sorkness R. L. Wenzel S. Hammel J. Curran-Everett D. Comhair S. A. (2010). Use of Exhaled Nitric Oxide Measurement to Identify a Reactive, At-Risk Phenotype Among Patients with Asthma. Am. J. Respir. Crit. Care Med. 181 (10), 1033–1041. 10.1164/rccm.200905-0695OC 20133930 PMC2874447

[B15] Eger K. Kroes J. A. Ten Brinke A. Bel E. H. (2021). Long-Term Therapy Response to Anti-IL-5 Biologics in Severe Asthma-A Real-Life Evaluation. J. Allergy Clin. Immunol. Pract. 9 (3), 1194–1200. 10.1016/j.jaip.2020.10.010 33069885

[B16] Fahy J. V. Boushey H. A. Lazarus S. C. Mauger E. A. Cherniack R. M. Chinchilli V. M. (2001). Safety and Reproducibility of Sputum Induction in Asthmatic Subjects in a Multicenter Study. Am. J. Respir. Crit. Care Med. 163 (6), 1470–1475. 10.1164/ajrccm.163.6.9901105 11371420

[B17] Farne H. A. Wilson A. Powell C. Bax L. Milan S. J. (2017). Anti-IL5 Therapies for Asthma. Cochrane Database Syst. Rev. 9 (9), CD010834. 10.1002/14651858.CD010834.pub3 28933516 PMC6483800

[B18] Fricker M. Gibson P. G. Powell H. Simpson J. L. Yang I. A. Upham J. W. (2019). A Sputum 6-gene Signature Predicts Future Exacerbations of Poorly Controlled Asthma. J. Allergy Clin. Immunol. 144 (1), 51–e11. 10.1016/j.jaci.2018.12.1020 30682452

[B19] Global Strategy for Asthma Management and Prevention (2021). Global Strategy for Asthma Management and Prevention. Available at: https://ginasthma.org/wp-content/uploads/2021/05/GINA-Main-Report-2021-V2-WMS (Accessed June 8, 2021).

[B20] Hammad H. Lambrecht B. N. (2021). The Basic Immunology of Asthma. Cell 184 (6), 1469–1485. 10.1016/j.cell.2021.02.016 33711259

[B21] Hanania N. A. Wenzel S. Rosén K. Hsieh H. J. Mosesova S. Choy D. F. (2013). Exploring the Effects of Omalizumab in Allergic Asthma: an Analysis of Biomarkers in the EXTRA Study. Am. J. Respir. Crit. Care Med. 187 (8), 804–811. 10.1164/rccm.201208-1414OC 23471469

[B22] Harvey E. S. Langton D. Katelaris C. Stevens S. Farah C. S. Gillman A. (2020). Mepolizumab Effectiveness and Identification of Super-responders in Severe Asthma. Eur. Respir. J. 55 (5), 1902420. 10.1183/13993003.02420-2019 32139455

[B23] Hastie A. T. Mauger D. T. Denlinger L. C. Coverstone A. Castro M. Erzurum S. (2021). Mixed Sputum Granulocyte Longitudinal Impact on Lung Function in the Severe Asthma Research Program. Am. J. Respir. Crit. Care Med. 203 (7), 882–892. 10.1164/rccm.202009-3713OC 33545021 PMC8017570

[B24] Hinks T. S. C. Brown T. Lau L. C. K. Rupani H. Barber C. Elliott S. (2016). Multidimensional Endotyping in Patients with Severe Asthma Reveals Inflammatory Heterogeneity in Matrix Metalloproteinases and Chitinase 3-like Protein 1. J. Allergy Clin. Immunol. 138 (1), 61–75. 10.1016/j.jaci.2015.11.020 26851968 PMC4929135

[B25] Holguin F. Cardet J. C. Chung K. F. Diver S. Ferreira D. S. Fitzpatrick A. (2020). Management of Severe Asthma: a European Respiratory Society/American Thoracic Society Guideline. Eur. Respir. J. 55 (1), 1900588. 10.1183/13993003.00588-2019 31558662

[B26] Jackson D. J. Busby J. Pfeffer P. E. Menzies-Gow A. Brown T. Gore R. (2021). Characterisation of Patients with Severe Asthma in the UK Severe Asthma Registry in the Biologic Era. Thorax 76 (3), 220–227. 10.1136/thoraxjnl-2020-215168 33298582 PMC7892381

[B27] Kavanagh J. E. d'Ancona G. Elstad M. Green L. Fernandes M. Thomson L. (2020). Real-World Effectiveness and the Characteristics of a "Super-responder" to Mepolizumab in Severe Eosinophilic Asthma. Chest 158 (2), 491–500. 10.1016/j.chest.2020.03.042 32275980

[B28] Kavanagh J. E. Hearn A. P. Dhariwal J. d'Ancona G. Douiri A. Roxas C. (2021). Real-World Effectiveness of Benralizumab in Severe Eosinophilic Asthma. Chest 159 (2), 496–506. 10.1016/j.chest.2020.08.2083 32882249

[B29] Korevaar D. A. Westerhof G. A. Wang J. Cohen J. F. Spijker R. Sterk P. J. (2015). Diagnostic Accuracy of Minimally Invasive Markers for Detection of Airway Eosinophilia in Asthma: a Systematic Review and Meta-Analysis. Lancet Respir. Med. 3 (4), 290–300. 10.1016/S2213-2600(15)00050-8 25801413

[B30] Kraft M. Brusselle G. FitzGerald J. M. Pavord I. D. Keith M. Fagerås M. (2021). Patient Characteristics, Biomarkers and Exacerbation Risk in Severe, Uncontrolled Asthma. Eur. Respir. J. 58 (6), 2100413. 10.1183/13993003.00413-2021 34112734

[B31] Kroes J. A. Zielhuis S. W. van Roon E. N. Ten Brinke A. (2020). Prediction of Response to Biological Treatment with Monoclonal Antibodies in Severe Asthma. Biochem. Pharmacol. 179, 113978. 10.1016/j.bcp.2020.113978 32305434

[B32] Malinovschi A. Fonseca J. A. Jacinto T. Alving K. Janson C. (2013). Exhaled Nitric Oxide Levels and Blood Eosinophil Counts Independently Associate with Wheeze and Asthma Events in National Health and Nutrition Examination Survey Subjects. J. Allergy Clin. Immunol. 132 (4), 821–825. 10.1016/j.jaci.2013.06.007 23890753

[B33] Malinovschi A. Janson C. Borres M. Alving K. (2016). Simultaneously Increased Fraction of Exhaled Nitric Oxide Levels and Blood Eosinophil Counts Relate to Increased Asthma Morbidity. J. Allergy Clin. Immunol. 138 (5), 1301–e2. 10.1016/j.jaci.2016.01.044 27113848

[B34] Matsunaga K. Kuwahira I. Hanaoka M. Saito J. Tsuburai T. Fukunaga K. (2021). An Official JRS Statement: The Principles of Fractional Exhaled Nitric Oxide (FeNO) Measurement and Interpretation of the Results in Clinical Practice. Respir. Investig. 59 (1), 34–52. 10.1016/j.resinv.2020.05.006 32773326

[B35] McDowell P. J. Diver S. Yang F. Borg C. Busby J. Brown V. (2021). The Inflammatory Profile of Exacerbations in Patients with Severe Refractory Eosinophilic Asthma Receiving Mepolizumab (The MEX Study): a Prospective Observational Study. Lancet Respir. Med. 9 (10), 1174–1184. 10.1016/S2213-2600(21)00004-7 33971168

[B36] Miller M. R. Hankinson J. Brusasco V. Burgos F. Casaburi R. Coates A. (2005). Standardisation of Spirometry. Eur. Respir. J. 26 (2), 319–338. 10.1183/09031936.05.00034805 16055882

[B37] Moore W. C. Hastie A. T. Li X. Li H. Busse W. W. Jarjour N. N. (2014). Sputum Neutrophil Counts Are Associated with More Severe Asthma Phenotypes Using Cluster Analysis. J. Allergy Clin. Immunol. 133 (6), 1557–e5. 10.1016/j.jaci.2013.10.011 24332216 PMC4040309

[B38] Nagase H. Adachi M. Matsunaga K. Yoshida A. Okoba T. Hayashi N. (2020). Prevalence, Disease burden, and Treatment Reality of Patients with Severe, Uncontrolled Asthma in Japan. Allergol. Int. 69 (1), 53–60. 10.1016/j.alit.2019.06.003 31311707

[B39] Nakamura Y. Tamaoki J. Nagase H. Yamaguchi M. Horiguchi T. Hozawa S. (2020). Japanese Guidelines for Adult Asthma 2020. Allergol. Int. 69 (4), 519–548. 10.1016/j.alit.2020.08.001 32893125

[B40] Oishi K. Hirano T. Suetake R. Ohata S. Yamaji Y. Ito K. (2017). A Trial of Oral Corticosteroids for Persistent Systemic and Airway Inflammation in Severe Asthma. Immun. Inflamm. Dis. 5 (3), 261–264. 10.1002/iid3.166 28474411 PMC5569377

[B41] Ortega H. G. Yancey S. W. Mayer B. Gunsoy N. B. Keene O. N. Bleecker E. R. (2016). Severe Eosinophilic Asthma Treated with Mepolizumab Stratified by Baseline Eosinophil Thresholds: a Secondary Analysis of the DREAM and MENSA Studies. Lancet Respir. Med. 4 (7), 549–556. 10.1016/S2213-2600(16)30031-5 27177493

[B42] Paggiaro P. L. Chanez P. Holz O. Ind P. W. Djukanović R. Maestrelli P. (2002). Sputum Induction. Eur. Respir. J. Suppl. 37, 3s–8s. 10.1183/09031936.02.00000302 12361361

[B43] Peters M. C. Dyjack N. Herrin R. Woodruff P. G. Rios C. O'Connor B. (2019). A Transcriptomic Method to Determine Airway Immune Dysfunction in T2-High and T2-Low Asthma. Am. J. Respir. Crit. Care Med. 199 (4), 465–477. 10.1164/rccm.201807-1291OC 30371106 PMC6376622

[B44] Price D. B. Rigazio A. Campbell J. D. Bleecker E. R. Corrigan C. J. Thomas M. (2015). Blood Eosinophil Count and Prospective Annual Asthma Disease burden: a UK Cohort Study. Lancet Respir. Med. 3 (11), 849–858. 10.1016/S2213-2600(15)00367-7 26493938

[B45] Ramonell R. P. Iftikhar I. H. (2020). Effect of Anti-IL5, Anti-IL5R, Anti-IL13 Therapy on Asthma Exacerbations: A Network Meta-Analysis. Lung 198 (1), 95–103. 10.1007/s00408-019-00310-8 31894410 PMC9136642

[B46] Reddel H. K. Taylor D. R. Bateman E. D. Boulet L. P. Boushey H. A. Busse W. W. (2009). An Official American Thoracic Society/European Respiratory Society Statement: Asthma Control and Exacerbations: Standardizing Endpoints for Clinical Asthma Trials and Clinical Practice. Am. J. Respir. Crit. Care Med. 180 (1), 59–99. 10.1164/rccm.200801-060ST 19535666

[B47] Rossios C. Pavlidis S. Hoda U. Kuo C. H. Wiegman C. Russell K. (2018). Sputum Transcriptomics Reveal Upregulation of IL-1 Receptor Family Members in Patients with Severe Asthma. J. Allergy Clin. Immunol. 141 (2), 560–570. 10.1016/j.jaci.2017.02.045 28528200

[B48] Salter B. M. Sehmi R. (2017). Hematopoietic Processes in Eosinophilic Asthma. Chest 152 (2), 410–416. 10.1016/j.chest.2017.01.021 28130045

[B49] Schleich F. Brusselle G. Louis R. Vandenplas O. Michils A. Pilette C. (2014). Heterogeneity of Phenotypes in Severe Asthmatics. The Belgian Severe Asthma Registry (BSAR). Respir. Med. 108 (12), 1723–1732. 10.1016/j.rmed.2014.10.007 25456708

[B50] Soma T. Iemura H. Naito E. Miyauchi S. Uchida Y. Nakagome K. (2018). Implication of Fraction of Exhaled Nitric Oxide and Blood Eosinophil Count in Severe Asthma. Allergol. Int. 67S, S3–S11. 10.1016/j.alit.2018.04.003 29754974

[B52] Takaku Y. Soma T. Uchida Y. Kobayashi T. Nakagome K. Nagata M. (2016). CXC Chemokine Superfamily Induced by Interferon-γ in Asthma: a Cross-Sectional Observational Study. Asthma Res. Pract. 2, 6. 10.1186/s40733-016-0021-y 27965774 PMC5142415

[B53] Tanaka A. Sato H. Akimoto K. Matsunaga T. Sagara H. (2021). Spontaneous Sputum Discriminates Inflammatory Phenotypes in Patients with Asthma. Ann. Allergy Asthma Immunol. 126 (1), 54–e1. 10.1016/j.anai.2020.06.017 32553777

[B54] Uchida Y. Soma T. Nakagome K. Kobayashi T. Nagata M. (2019). Implications of Prostaglandin D2 and Leukotrienes in Exhaled Breath Condensates of Asthma. Ann. Allergy Asthma Immunol. 123 (1), 81–e1. 10.1016/j.anai.2019.04.008 30986547

[B56] Watanabe H. Shirai T. Hirai K. Akamatsu T. Nakayasu H. Tamura K. (2021). Blood Eosinophil Count and FeNO to Predict Benralizumab Effectiveness in Real-Life Severe Asthma Patients. J. Asthma 17, 1–9. 10.1080/02770903.2021.1963769 34348060

[B57] Zeiger R. S. Schatz M. Li Q. Chen W. Khatry D. B. Gossage D. (2014). High Blood Eosinophil Count Is a Risk Factor for Future Asthma Exacerbations in Adult Persistent Asthma. J. Allergy Clin. Immunol. Pract. 2 (6), 741–750. 10.1016/j.jaip.2014.06.005 25439366

