# Supplementary figures and images for: Treatment Resistance in Severe Asthma Patients With a Combination of High Fraction of Exhaled Nitric Oxide and Low Blood Eosinophil Counts
Source: Front Pharmacol. 2022 Apr 20;13:836635. doi: 10.3389/fphar.2022.836635 (PMC9065285; doi:10.3389/fphar.2022.836635)

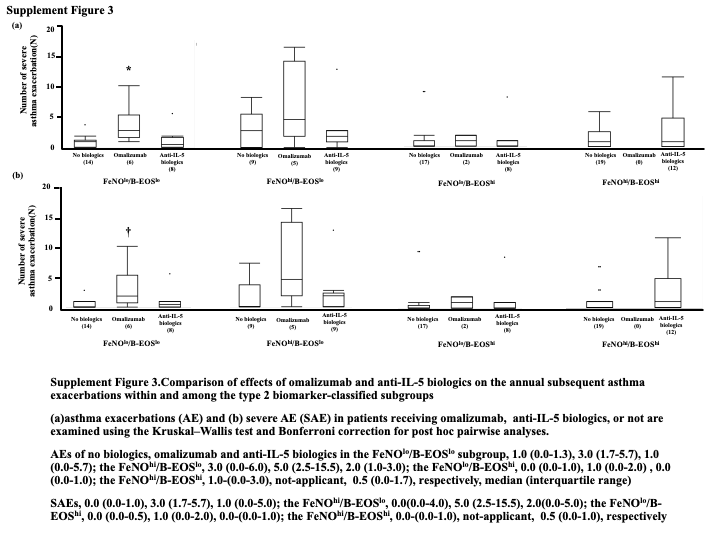

Supplement: Supplementary file 1 [file Image3.tiff]

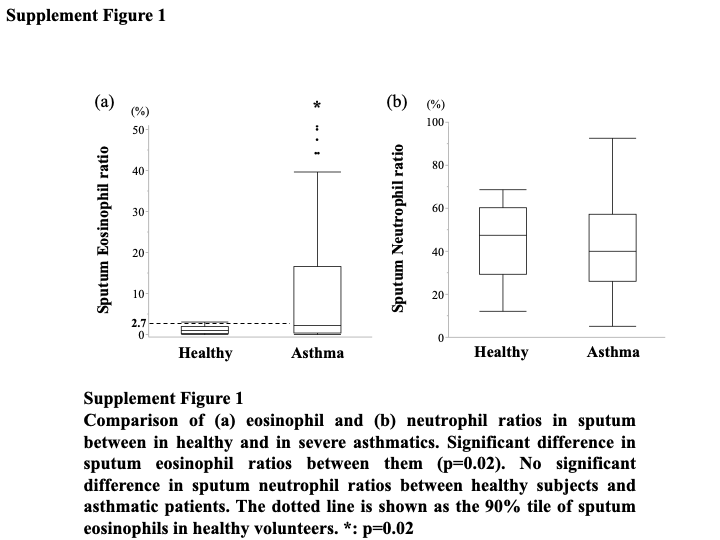

Supplement: Supplementary file 2 [file Image1.TIFF]

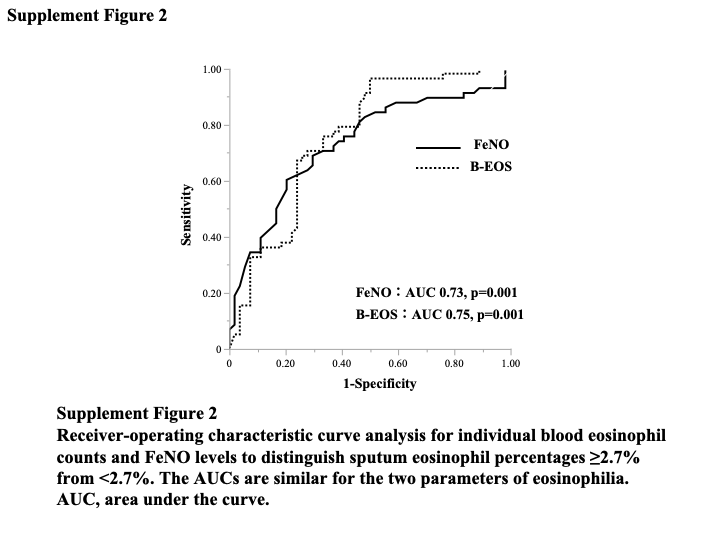

Supplement: Supplementary file 4 [file Image2.TIFF]
