# Supplementary material for: Treatment Resistance in Severe Asthma Patients With a Combination of High Fraction of Exhaled Nitric Oxide and Low Blood Eosinophil Counts
Source: Front Pharmacol. 2022 Apr 20;13:836635. doi: 10.3389/fphar.2022.836635 (PMC9065285; doi:10.3389/fphar.2022.836635)
Supplement: Supplementary file 3 [file DataSheet1.PDF]

Supplemental table 1 Demographics and clinical characteristics of severe asthmatics without baseline biologics in the subgroups classified by type 2 biomarker.

	**FeNO^{lo} B-EOS^{lo}**	**FeNO^{hi} B-EOS^{lo}**	**FeNO^{lo} B-EOS^{hi}**	**FeNO^{hi} B-EOS^{hi}**	**P value**
**N (%)**	26 (25.3)	19 (18.4)	28 (27.2)	30 (29.1)	
**Age, years**	64.0 (45.7-72.0)	57.0 (49.0-73.0)	63.0 (55.7-73.5)	66.5 (54.0-73.2)	NS
**Female/male, n(%)**	19 (73.1) /7 (26.9)	5 (26.3) /14 (73.7)	15 (53.5) /13 (46.4)	17 (56.7) /13 (43.3)	NS
**BMI, kg/m^2**	23.3 (20.6-24.5)	25.4 (22.0-27.3)	23.3 (21.9-24.8)	22.0 (20.0-23.7)	NS
**Smoking history, no/past/present, n (%)**	17 (65.3) / 9 (34.7) / 0 (0)	12 (63.2) / 5 (26.3) / 2 (10.5)	16 (57.1) / 10 (35.8) / 2 (7.1)	18 (60.0) / 9 (30.0) / 3 (10.0)	NS
**Duration of asthma (y)**	10 (4-25)	15 (4-31)	14 (8-18)	14 (4-24)	NS
**Allergic rhinitis, n (%)**	15 (57.7)	12 (63.1)	15 (53.5)	13 (43.3)	NS
**Chronic sinusitis, n (%)**	3 (11.5)	2 (10.5)	8 (28.5)	10 (33.3)	NS
**Atopic dermatitis, n (%)**	4 (15.3)	2 (10.5)	3 (10.7)	3 (10.0)	NS
**Urticaria, n (%)**	2 (7.7)	1 (5.3)	1 (3.5)	0 (0)	NS
**Previous annual number of asthma exacerbations**	1 (0-4)	3 (1-6)	1 (0-2)	1 (0-3)	NS
**LABA/LAMA/LTRA/Theophylline, n(%)**	23 (88.5) / 6 (23.1) 22 (84.6) /17 (65.4)	19 (100) / 9 (47.4) 16 (84.2) / 14 (73.7)	28 (100) / 6 (21.4) 26 (92.9) / 18 (64.2)	29 (96.7) / 13 (43.3) 26 (86.7) / 22 (73.3)	NS
**ICS dose (µg/d)**	1000 (800-1050)	1000 (800-1050)	1000 (800-1000)	900 (800-1000)	NS
**Oral corticosteroids, n (%)**	12 (46.1)*,†	8 (42.1)	5 (17.8)	6 (20.0)	**0.04**
**Oral corticosteroids, mg/day**	0 (0-5)	0 (0-5)	0 (0-5)	0 (0-0)	NS
**Biologics after the entry, n (%)**	12 (46.1)	10 (52.6)	11 (39.3)	11 (33.7)	NS
**Log total IgE, IU/L**	2.1 (1.8-2.3)	2.8 (2.2-3.2)††,‡	2.3 (1.8-2.7)	2.4 (2.1-2.9)‡‡	**0.006**
**Blood eosinophil count, /µL**	88 (47-181)	153 (68-208)	484 (331-692) ‡‡‡,§	625 (485-1098) ††,‡‡‡,§	**<0.0001**
**FeNO, ppb**	11 (6-17)	84 (42-133) ‡‡‡	18 (11-21)‡‡‡	85 (40-119) †††,‡‡‡	**<0.0001**
**Sputum eosinophil ratio, %**	0.2 (0-1.6)	4.6 (1.8-13.8)‡‡‡	5.3 (0.3-23.8) ‡	14.3 (1.9-29.7) ‡‡‡	**<0.0001**
**Sputum neutrophil ratio, %**	53.8 (40.1-69.1)	31.0 (23.3-65.3)	39.1 (18.7-51.2)	35.1 (25.5-51.0)	NS

**FEV_1, % of predicted**	84.2 (18.9)	86.2 (21.0)	80.0 (24.1)	81.5 (19.8)	NS
**FVC, % of predicted**	95.1 (19.7)	93.1 (16.6)	85.0 (25.9)	89.5 (18.4)	NS
**FEV_1/FVC, %**	72.3 (18.2)	67.8 (13.3)	69.1 (13.7)	64.9 (12.4)	NS

Significant p values are shown in boldface. Parametric data are expressed as mean (SD); nonparametric data are expressed as median (25%–75%). Oral corticosteroid doses are e expressed as median (min-max). ANOVA and the Kruskal–Wallis test were performed for parametric continuous variables and nonparametric variables, respectively. Categorical variables were tested the χ^2 test or the Fisher exact test, as appropriate. Further exploration of the results with significant differences in the initial analyses was performed by post hoc pairwise analyses with Bonferroni correction or by the Mann–Whitney U-test, as appropriate.

$FeNO^{lo}$, low FeNO; $FeNO^{hi}$, high FeNO; $B\text{-}EOS^{lo}$, low B-EOS; $B\text{-}EOS^{hi}$, high B-EOS; LABA, long-acting b2- agonist; LTRA, leukotriene receptor antagonist; NA, not applicable.

*p=0.03; vs $FeNO^{hi}$/$B\text{-}EOS^{hi}$; †p=0.02, ††p=0.04, †††p<.0001; vs $FeNO^{lo}$/ $B\text{-}EOS^{hi}$; ‡p=0.002, ‡‡p=0.006, ‡‡‡p<.0001; vs $FeNO^{lo}$/ $B\text{-}EOS^{lo}$; §p<.0001; vs $FeNO^{hi}$/$B\text{-}EOS^{lo}$.
